# RACK-1 overexpression protects against goniothalamin-induced cell death

**DOI:** 10.1016/j.toxlet.2009.08.012

**Published:** 2009-12-15

**Authors:** S.H. Inayat-Hussain, L.T. Wong, K.M. Chan, N.F. Rajab, L.B. Din, R. Harun, A. Kizilors, N. Saxena, M. Mourtada-Maarabouni, F. Farzaneh, G.T. Williams

**Affiliations:** a Toxicology and Biocompatibility Laboratory, Faculty of Allied Health Sciences, Universiti Kebangsaan Malaysia, Jalan Raja Muda Abdul Aziz, 50300 Kuala Lumpur, Malaysia; b Faculty of Science and Technology, Universiti Kebangsaan Malaysia, Bangi 43600, Selangor DE, Malaysia; c UKM Medical Molecular Biology Institute, Universiti Kebangsaan Malaysia, Bandar Tun Razak, 56000 Kuala Lumpur, Malaysia; d King's College London, Department of Haematological Medicine, The Rayne Institute 123, Coldharbour Lane, London SE5 9NU, UK; e Institute for Science and Technology in Medicine and School of Life Sciences, Keele University, Keele ST5 5AZ, UK

**Keywords:** Goniothalamin, RACK-1, DNA damage, Cell death

## Abstract

Goniothalamin, a styryllactone, has been shown to induce cytotoxicity via apoptosis in several tumor cell lines. In this study, we have examined the potential role of several genes, which were stably transfected into T-cell lines and which regulate apoptosis in different ways, on goniothalamin-induced cell death. Overexpression of full-length receptor for activated protein C-kinase 1 (RACK-1) and pc3n3, which up-regulates endogenous RACK-1, in both Jurkat and W7.2 T cells resulted in inhibition of goniothalamin-induced cell death as assessed by MTT and clonogenic assays. However, overexpression of rFau (antisense sequence to *F*inkel–Biskis–Reilly murine sarcoma virus-*a*ssociated *u*biquitously expressed gene) in W7.2 cells did not confer resistance to goniothalamin-induced cell death. Etoposide, a clinically used cytotoxic agent, was equipotent in causing cytotoxicity in all the stable transfectants. Assessment of DNA damage by Comet assay revealed goniothalamin-induced DNA strand breaks as early as 1 h in vector control but this effect was inhibited in RACK-1 and pc3n3 stably transfected W7.2 cells. This data demonstrate that RACK-1 plays a crucial role in regulating cell death signalling pathways induced by goniothalamin.

## 1 Introduction

Given the molecular complexity of the cell death processes, approaches for therapeutic intervention include development of small molecules from either combinatorial chemistry or from natural products to modulate death regulators. Clearly, natural products serve as an important source of chemical structural diversity and have been the mainstay of the discovery of new leads for cytotoxic drugs in the past 25 years (Cragg and Newman, 2003).

Styryllactones are a group of secondary metabolites found ubiquitously in Goniothalamus plant species. To date, there are approximately 100 styryllactones, including synthetic analogs and some of these compounds have been demonstrated to show pharmacological properties such as anticancer, anti-inflammation, immunosuppressive and antiparasitic activities (de Fátima et al., 2006a). Since styryllactones have structural similarity with stilbenes and the fact that many stilbenes have been patented for drug development (Inayat-Hussain and Thomas, 2004), it is envisaged that there will be an increase in drug development of novel anticancer compounds incorporating stilbene and lactone moieties in the future. In this respect, goniothalamin (GTN) a plant styryllactone isolated from *Goniothalamus andersonii*, induces cytotoxicity in a variety of cancer cell lines including cervical (Hela), gastric (HGC-27), kidney (786-0), breast carcinomas (MCF7, T47D and MDA-MB-231) and leukemia (HL-60, Jurkat and CEM-SS) (Ali et al., 1997; Inayat-Hussain et al., 1999, 2003; Pihie et al., 1998; Rajab et al., 2005; Chen et al., 2005; de Fátima et al., 2006b). Our group and others have demonstrated that cytotoxicity of GTN in human leukemia (HL-60 and Jurkat) and human breast cancer cells (MDA-MB-231) occurs via apoptotic cell death (Inayat-Hussain et al., 1999, 2003; Chen et al., 2005). Intracellular redox status disruption and DNA damage are early signals for cell death prior to loss of mitochondrial membrane potential and activation of the apical caspase 9. However, the precise molecular regulation of cell death induced by goniothalamin is not fully understood.

Advances in cDNA library based functional expression cloning have led to the discovery of novel genes which regulate cell death through apoptosis (Williams and Farzaneh, 2004). Our group and others have successfully identified novel apoptotic controlling genes including LUCA15/RBM5 a novel tumor suppressor gene (Sutherland et al., 2000), fau (*F*inkel–Biskis–Reilly murine sarcoma virus (FBR-MuSV) *a*ssociated ubiquitously expressed gene) (Mourtada-Maarabouni et al., 2003), vATPase (Anderson and Williams, 2003), protein phosphatase 4 (PP4) (Mourtada-Maarabouni et al., 2003), Toso (Hitoshi et al., 1998) and RACK-1 (Mourtada-Maarabouni et al., 2005). It has been shown that the fau antisense sequence (rFau) suppresses apoptosis induced by cisplatin, ultraviolet (UV) radiation and dexamethasone. Similarly, cells overexpressing the partial sequence of PP4 (4n10) are resistant to the induction of cell death by dexamethasone and UV radiation (Mourtada-Maarabouni et al., 2003).

In recent years, accumulating evidence has demonstrated that several kinase signalling cascades can result in regulated cell death (Horbinski and Chu, 2005). Amongst these, the protein kinase C family, which consists of multiple isozymes, plays a pivotal role in cell death, cell growth and differentiation (Fields and Murray, 2008). Localisation of PKCs to specific cellular compartments involves the receptor for activated C-kinase 1 (RACK-1). RACK-1, a multiple WD motif-containing protein, is important in regulating cell growth by suppressing apoptosis (Mourtada-Maarabouni et al., 2005). Choi et al. (2003) have shown that RACK-1 gene expression is induced by NF-κB leading to cell survival of PC-12 cells upon withdrawal of nerve growth factor. Consistent with this, RACK-1 overexpression relieves EIA-mediated growth inhibition, protects SAOS-2 tumor cells (Sang et al., 2001) and produces resistance to dexamethasone and UV-induced apoptosis in W7.2 thymoma cells (Mourtada-Maarabouni et al., 2005).

Since RACK-1, rFau and 4n.10 result in cell survival induced by DNA damaging agents including cisplatin and UV radiation, therefore it will be important to investigate the effects of GTN which also induces DNA damage leading to cytotoxicity. To test the hypothesis that GTN-induced cytotoxicity is regulated by RACK-1, rFau and 4n.10, we investigated the effects of GTN on a panel of T-cell lines with specific blocks to the induction of cell death. These human and mouse T-cell clones overexpress RACK-1, pc3n3 (partial sequence of RACK-1), rFau and 4n.10. This study demonstrates that only overexpression of RACK-1 and pc3n3 protects against goniothalamin-induced cell death.

## 2 Materials and methods

### 2.1 Chemicals

GTN was obtained as described previously (Chan et al., 2006). Etoposide was purchased from Sigma–Aldrich.

### 2.2 Cell culture

The apoptosis-sensitive clone W7.2, originally derived from mouse thymoma line WEHI-105.726 (Longthorne and Williams, 1997), and the cloned human leukaemic cell line Jurkat.JKM1 were maintained in RPMI-1640 medium (Sigma) supplemented with 10% heat inactivated fetal calf serum (Hyclone), 2 mM L-glutamine and 200 μg/ml gentamycin (Sigma), at 37 °C in a 5% CO_2_ humidified incubator.

### 2.3 Establishment of stable clones

The generation of the constructs pcDNA3.1/pc3n3, pcDNA3/rfau and pcDNA3/4n.10 in expression vectors and the establishment of stable clones expressing these constructs have been previously described (Mourtada-Maarabouni et al., 2004, 2005). The production of stable transfectants of RACK-1 (1dbEST Id: 13833312, GeneBank Acc: BU515312; IMAGE: 6511980) has been described in Mourtada-Maarabouni et al. (2005).

### 2.4 Clonogenic assay

Survival of control and treated cells was also assessed by the ability of the cells to form colonies in soft agar (based on Longthorne and Williams, 1997). Essentially, 1 × 10^3^ Jurkat cells and 5 × 10^4^ W7.2 cells were treated with 5 μM GTN. An equal proportion of the culture from each experimental condition was diluted in 5 ml Iscove's medium (Sigma) containing 20% heat inactivated fetal calf serum, 10% W7.2- or Jurkat-conditioned medium and 0.3% Noble agar (Difco) and plated in 60 mm dishes. Dishes were also overlaid with 2.5 ml Iscove's complete medium containing 10% cell-conditioned medium. The cells were left for 16 days incubation at 37 °C in 5% CO_2_ and the number of colonies formed was counted.

### 2.5 Western blot analysis

Samples (1 × 10^6^ cells) were mixed with Laemmli's loading buffer and subjected to 10% SDS-PAGE and transferred onto a PVDF membrane. The blots were then probed with the anti-RACK-1 (1:2500 dilution; clone 20, BD Transduction Laboratories, #R20620), or anti-β-actin antibody (dilution 1:5000; Sigma, #A5441), followed by the appropriate horseradish peroxidase-conjugated secondary antibodies. The secondary antibodies used were anti–mouse IgM (diluted 1:1000; Santa Cruz, #sc2064) and anti-mouse immunoglobulin (diluted 1:800; Dakko, #P0447).

### 2.6 Alkaline Comet assay

The Comet assay was carried out as described previously (Rajab et al., 2005). Briefly, cells (1 × 10^6^ cells/ml) were treated in 6 well plates with 5 μM GTN for 1 h. Analysis was carried out with a fluorescence microscope and DNA damage was assessed in 50 cells where tail moment was quantified using the Comet Assay III (Perceptive Instrument Ltd).

### 2.7 MTT assay

Cytotoxicity of GTN was evaluated using the MTT assay. Based on our previous study, a range of concentrations (from 5 to 150 μM) was used and these concentrations were demonstrated to exhibit a concentration-dependent increase of cell death in Jurkat T cells (Inayat-Hussain et al., 1999). Briefly, cells were treated at 1 × 10^6^ cells/ml with final concentrations of 12.5, 25, 50 and 100 μM GTN. After 24 h incubation, 20 μl of 5 mg/ml MTT (Sigma) solution was added to each well and further incubated for 4 h at 37 °C. Subsequently, 150 μl medium was discarded from each well before adding 150 μl DMSO (Fischer Scientific). The cytotoxic effects of GTN were detected by measuring the absorbance of each well at 570 nm and calculated as described previously (Chen et al., 2005).

### 2.8 Statistical analysis

The data were presented as the means ± standard error of the means (S.E.M.). Statistical analysis was performed with Student's *t*-test for comparison between two means. One-way ANOVA with Tukey post hoc test was used to test the significance between multiple means. A *p*-value of <0.05 was considered statistically significant.

## 3 Results

### 3.1 Characterization of GTN-induced cytotoxicity in apoptosis-resistant cell lines

**Fig. 1:**
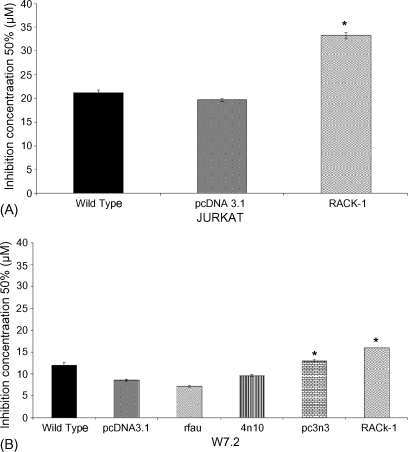
The IC_50_s of GTN treated Jurkat cells (panel A) and W7.2 cells (panel B). Cells (1 × 10^6^ cells/ml) were treated with various concentrations of GTN (0–100 μM) for 24 h before cytotoxicity was evaluated using MTT assay. Cumulative data represent the mean ± SEM of three separate experiments (*p < 0.05 against vector control).

To investigate the effects of GTN on cells expressing known apoptosis-resistance genes, stably transfected Jurkat and W7.2 T-cells were employed. Cytotoxicity was assessed using the MTT assay and the IC_50_ of GTN in the various cells after 24 h treatment was determined as shown in Fig. 1A. The IC_50_ for wt Jurkat was 21 ± 1 μM which was similar to the IC_50_ for the vector control (20 ± 0.5 μM). In contrast, there was a significant increase in the IC_50_ of RACK-1 overexpressing Jurkat cells (33 ± 1 μM).

In order to confirm the observation in the Jurkat cells, a panel of W7.2 T cell lines was also employed (Fig. 1B). GTN treatment of wt W7.2 cells resulted in cytotoxicity with an IC_50_ of 12 ± 1 μM confirming that these mouse cells were more sensitive than human Jurkat cells. In agreement with the Jurkat RACK-1 data, GTN treatment of W7.2 cells overexpressing RACK-1 (IC_50_ = 16 ± 0.1 μM) revealed resistance to GTN compared to the IC_50_ of the vector control cells (8.5 ± 0.3 μM). Resistance to cytotoxicity was also observed in cells transfected with pc3n3, which increases endogenous RACK-1 levels (Mourtada-Maarabouni et al., 2005), where the IC_50_ was 13 ± 0.5 μM. However, GTN cytotoxicity was not affected in rFau and 4n10 stable transfectants where the IC_50_ values were 7 ± 0.3 and 10 ± 0.4 μM, respectively.

**Fig. 2:**
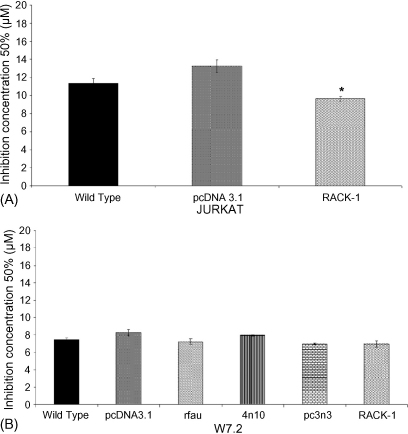
The IC_50_s of etoposide treated Jurkat cells (panel A) and W7.2 cells (panel B). Cells (1 × 10^6^ cells/ml) were treated with various concentrations of etoposide (0–100 μM) for 24 h before cytotoxicity was evaluated using MTT assay. Cumulative data represent the mean ± SEM of three separate experiments (*p < 0.05 against vector control).

To confirm the specificity of the effects observed with GTN, we analysed the effects of topoisomerase II inhibitor etoposide in both panels of cells. As shown in Fig. 2A, etoposide treated RACK-1 overexpressing Jurkat cells showed an IC_50_ of 10 ± 0.4 μM which is lower than the IC_50_ of the vector control (13 ± 1 μM) confirming no resistance by RACK-1. Similarly, treatment with etoposide did not reveal any reduction in cytotoxicity in the RACK-1 overexpressing W7.2 cells, where the IC_50_ was 7 ± 0.7 μM compared to the vector control cells (8 ± 0.6 μM). Furthermore, rFau overexpressing W7.2 cells did not show any increased cytotoxicity to etoposide treatment (Fig. 2B). Since significant inhibition of GTN cytotoxicity was only observed in pc3n3 and RACK-1 stable transfectants, further experiments were performed only in these cells.

### 3.2 Clonogenic assessment using soft agar colony formation

**Fig. 3:**
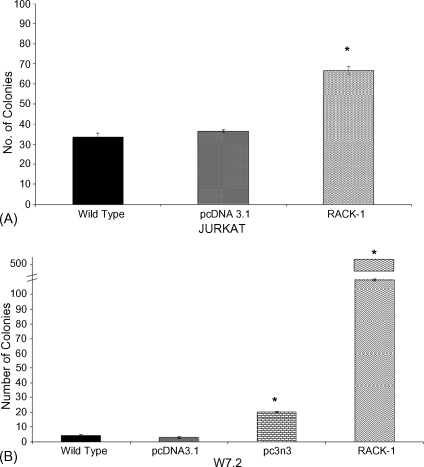
Clonogenic assessment following GTN treatment on Jurkat cells (panel A) and W7.2 cells (panel B). 1 × 10^3^ Jurkat cells and 5 × 10^4^ W7.2 cells were treated with 5 μM GTN and the cells were left for a 16-day incubation. Data represent mean ± SEM from three independent experiments (*p < 0.05 against vector control).

**Fig. 4:**
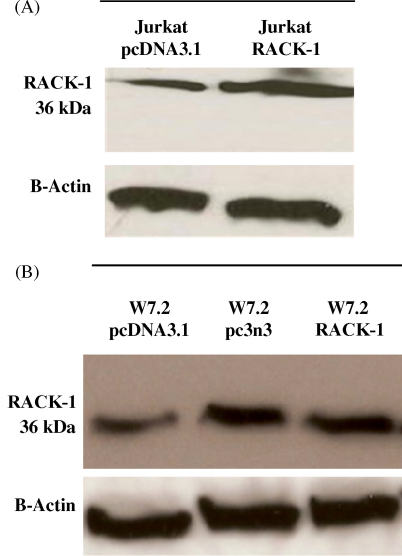
Protein expression of RACK-1 in Jurkat and W7.2 cells. Western blotting of RACK-1 protein in (a) Jurkat vector control (pcDNA3.1) and Jurkat RACK-1 and (b) W7.2 vector control (pcDNA3.1), W7.2-pc3n3 and W7.2 RACK-1 transfected clones. The corresponding Western blots with anti-β-actin antibody are shown to demonstrate equivalent loadings.

To ascertain the colony-forming ability of GTN treated cells, clonogenic assessment of a panel of Jurkat and W7.2 stable transfectants was carried out in soft agar. Selection of GTN concentration was based on preliminary studies using 2.5–10 μM GTN (data not shown). Colony-forming ability was best assessed with 5 μM GTN treatment for 16 days as shown in Fig. 3A. In agreement with the MTT cytotoxicity data (see Fig. 1A), the clonogenic assay also confirmed that RACK-1 protected against cytotoxicity induced by GTN in both Jurkat and W7.2 cells. There was an increase of 30 colonies in Jurkat RACK-1 cells over vector control cells and very significant protection was detectable in W7.2 RACK-1 cells where more than 500 colonies were observed in the presence of GTN (Fig. 3B). In addition, the partial sequence of RACK-1 (pc3n3) also protected against GTN-induced cytotoxicity in W7.2 cells where more than 17 colonies were observed, further strengthening the critical role of RACK-1 in the induction of cell death by GTN. Immunoblotting of RACK-1 protein expression in W7.2 and Jurkat cells confirmed the overexpression of RACK-1 in the stable transfectants expressing the full sequence of RACK-1 or its partial sequence (pc3n3) (Fig. 4).

### 3.3 Assessment of DNA damage by Comet assay

**Fig. 5:**
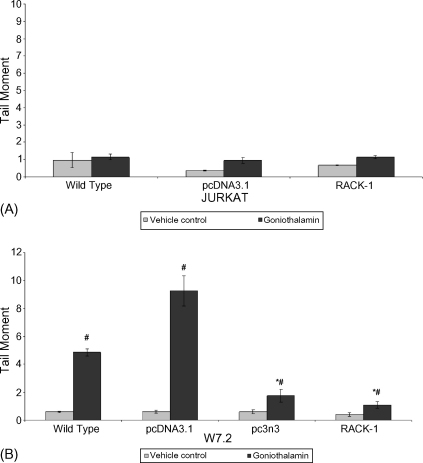
DNA damaging effects of GTN treated Jurkat cells (panel A) and W7.2 (panel B). 1 × 10^6^ cells were treated with 5 μM GTN for 1 h prior to Comet analysis. Data represent mean ± SEM from three independent experiments (*p < 0.05 against vector control, ^#^*p* < 0.05 against vehicle control).

GTN has been demonstrated to cause DNA damage in leukemia cell lines (HL-60 and CEM-SS), MDA-MB231 breast cancer cells and vascular smooth muscle cells (Rajab et al., 2005; Chan et al., 2006; Umar-Tsafe et al., 2004). In this study, the alkaline Comet assay was used to determine whether GTN could induce DNA damage in the panel of T cells. As shown in Fig. 5A, GTN did not induce DNA damage in wt, vector control and RACK-1 overexpressing Jurkat cells at concentrations used in the soft agar cloning assay (5 μM). However, in W7.2 cells, GTN did induce DNA damage in the wt and more significantly in the vector control cells (Fig. 5B). Interestingly, in the panel of W7.2 cells overexpressing the partial sequence to RACK-1 (pc3n3) and RACK-1, no DNA damage was observed, suggesting a protection by these genes.

## 4 Discussion

Using a panel of T-cell lines where the cell death pathway has been genetically inhibited at specific stages, we tested whether GTN's toxicity was affected in these cells. Our data demonstrate that GTN cytotoxicity was not inhibited by rFau stable transfectants although overexpression of rFau suppresses cell death induced by UV radiation and cisplatin in W7.2 cells (Mourtada-Maarabouni et al., 2004). Similarly, etoposide treated rFau overexpressing W7.2 cells did not show any resistance to cell death as compared to the vector control cells. It is interesting to note that Bcl-2 blocks cell death induced by the expression of fau in the sense orientation (Mourtada-Maarabouni et al., 2004). Fau contains a ubiquitin-like polypeptide called FUBI which has been shown to bind Bcl-G. This is consistent with a recent study by Wang et al. (2008) who demonstrated that cisplatin down-regulates Bcl-2 by dephosphorylation and ubiquitination leading to its proteosomal degradation. It will be interesting to investigate whether Bcl-2 plays a role in GTN cytotoxicity.

4n.10, the partial sequence of PP4 which is a member of the PP2A family of serine/threonine protein phosphatases down-regulates endogenous PP4 resulting in cell death (Mourtada-Maarabouni et al., 2003). Emerging evidence indicates that PP4 may play important and complex roles in apoptosis and cell proliferation especially in T-cells (Mourtada-Maarabouni and Williams, 2008, 2009). Since GTN-induced cytotoxicity was not affected when PP4 was down-regulated in 4n.10 stable transfectants, this result implies that PP4 may not regulate cell survival or death induced by this natural product.

**Fig. 6:**
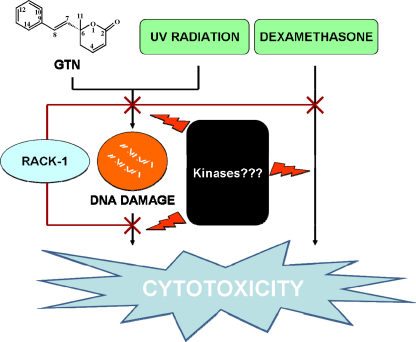
Schematic presentation of RACK-1 regulation in GTN-induced cytotoxicity. RACK-1 has also been demonstrated to regulate UV radiation and dexamethasone induced cytotoxicity. However, the effects of specific kinases in this model is yet to be identified.

Our data demonstrate that overexpression of RACK-1 in Jurkat and W7.2 cells protected against GTN-induced cell death as assessed by both MTT and soft agar colony-forming assays. RACK-1 is one of the adaptor proteins involved in molecular signalling by binding to protein kinase C, leading to its activation (Schechtman and Mochly-Rosen, 2001). This multiple WD-motif-containing protein has been demonstrated to activate JNK via PKC, thus promoting melanoma growth in nude mice (Lopez-Bergami et al., 2005). RACK-1 also rescues adenoviral EIA-induced SAOS-2 cells apoptosis (Sang et al., 2001). Binding of RACK-1 to p73 but not p53 has been demonstrated to prevent apoptosis in SAOS-2 cells (Ozaki et al., 2003). p73 functions in a pathway mediating p53-independent cell death produced by cytotoxic drugs especially in p53-null cells such as Jurkat (Karpinich et al., 2006). Indeed, etoposide treatment of Jurkat cells causes an increase in phosphorylation of p73, resulting in apoptosis as early as 45 min after treatment (Karpinich et al., 2006). Similarly, another genotoxic stress-inducing agent, cis-platinum results in p73 elevation in CHO cells which is inhibited by RACK-1 (Mor et al., 2008). Therefore it might be expected that RACK-1 overexpressing Jurkat cells would also be resistant to etoposide as RACK-1 can inactivate p73. However, our study shows no difference in the IC_50_ of etoposide in RACK-1 overexpressing Jurkat and W7.2 cells compared to the vector control cells. These intriguing results may be attributed to the possible interaction of pRB which has been demonstrated to physically bind RACK-1, thus diminishing the RACK-1-dependent inhibition of p73 (Ozaki et al., 2003). Taken together, current findings on RACK-1 suggest that this protein can rescue cells from the effects of only some genotoxic agents. Similar to UV radiation and dexamethasone (Mourtada-Maarabouni et al., 2005), evidence from our current work demonstrates that, in addition to RACK-1, pc3n3, which up-regulates endogenous RACK-1, also confer resistance to GTN-induced cytotoxicity in Jurkat and W7.2 cells (Fig. 6). It is interesting to note that the type of DNA damage may play a role in resistance to RACK-1. Goniothalamin, unlike etoposide, does not inhibit topoisomerase II (data not shown). As GTN has been shown to modulate the redox status in MDA-MB 231 cells (Chen et al., 2005), it is tempting to speculate that highly reactive intermediates of GN such as epoxide derivatives (probably goniothalamin epoxide) from phase I xenobiotic metabolism may occur and can also conjugate to GSH and damage the DNA via adduction. The molecular cross-talk between RACK-1 and other putative proteins leading to inhibition of DNA damage and cytotoxicity warrants further investigation. Although it is possible that RACK-1 expression could protect cells from GTN-induced suppression of proliferation, this is likely to be a relatively minor component since firstly the very substantial protective effects of RACK-1 in the colony-forming assay was produced at 5 μM, a concentration which has only a small effect on proliferation (data not shown). Secondly, colonies appear at the same rate in plates containing cells with and without RACK-1.

In conclusion, our data demonstrate that overexpression of RACK-1 and pc3n3 which upregulates the endogenous RACK-1, protects against GTN-induced cell death leading to increased cell survival.
